# Cortical white matter: no longer a silent partner

**DOI:** 10.3389/fnana.2025.1726067

**Published:** 2026-01-02

**Authors:** Kathleen S. Rockland, R. Jarrett Rushmore

**Affiliations:** Department of Anatomy and Neurobiology, Boston University Chobanian & Avedisian School of Medicine, Boston, MA, United States

**Keywords:** cytoskeleton, endpoints, holistic, myelination, topographic, white matter, tracts, axon bundle

## Abstract

This takes the position that the cell-sparse cortical white matter (WM) of gyrencephalic brains has too long held a secondary place in neuroanatomical investigations of cell-dense gray matter (GM) regions. This is unjustified and even problematic because WM, like GM, has its own subcellular, cellular, and supracellular multi-scale organization. Axons are not passive cables or wires, but engage in multiple processes, some in cooperation with neurons in the GM and, as increasingly recognized, also inter- and intra-axonal. In five sections of this review, we revisit traditional assumptions about WM organization and touch on recent results regarding: the axonal cytoskeleton and myelination, neuroanatomical approaches to global WM organization, open issues about “endpoints” (i.e., origin and termination of axon bundles), and orderly vs. “scrambled” topographies. There has been significant research progress at all spatial scales, and there is good reason to anticipate a more holistic approach in the next stages that will bring WM investigations more in line with the integrative approaches already customary in GM investigations.

## Introduction

As recognized already in the 1600' s, the large expanse of cortical white matter (WM), seemingly uniform at first glance, is not amorphous (see quotations from Malpighi, Steno, and Willis in chapter 2 in Schmahmann and Pandya, 2006). Indeed, as has been well documented at the anatomical level, distinguishable bundles of fiber tracts are consistently identified by multiple techniques, from gross dissection to high-resolution imaging. The full complexity of the WM, however, remains a subject of active research, as does the nature of its cooperative interactions with gray matter (GM). Whereas WM has been commonly viewed in its capacity as a conduction" compartment, forwarding "information" between pre- and postsynaptic cellular GM endpoints, there is now greater recognition that the WM supports multiple communication modes and dynamic processes, at both the anatomical micro- and macro-scales.

**Figure 1:**
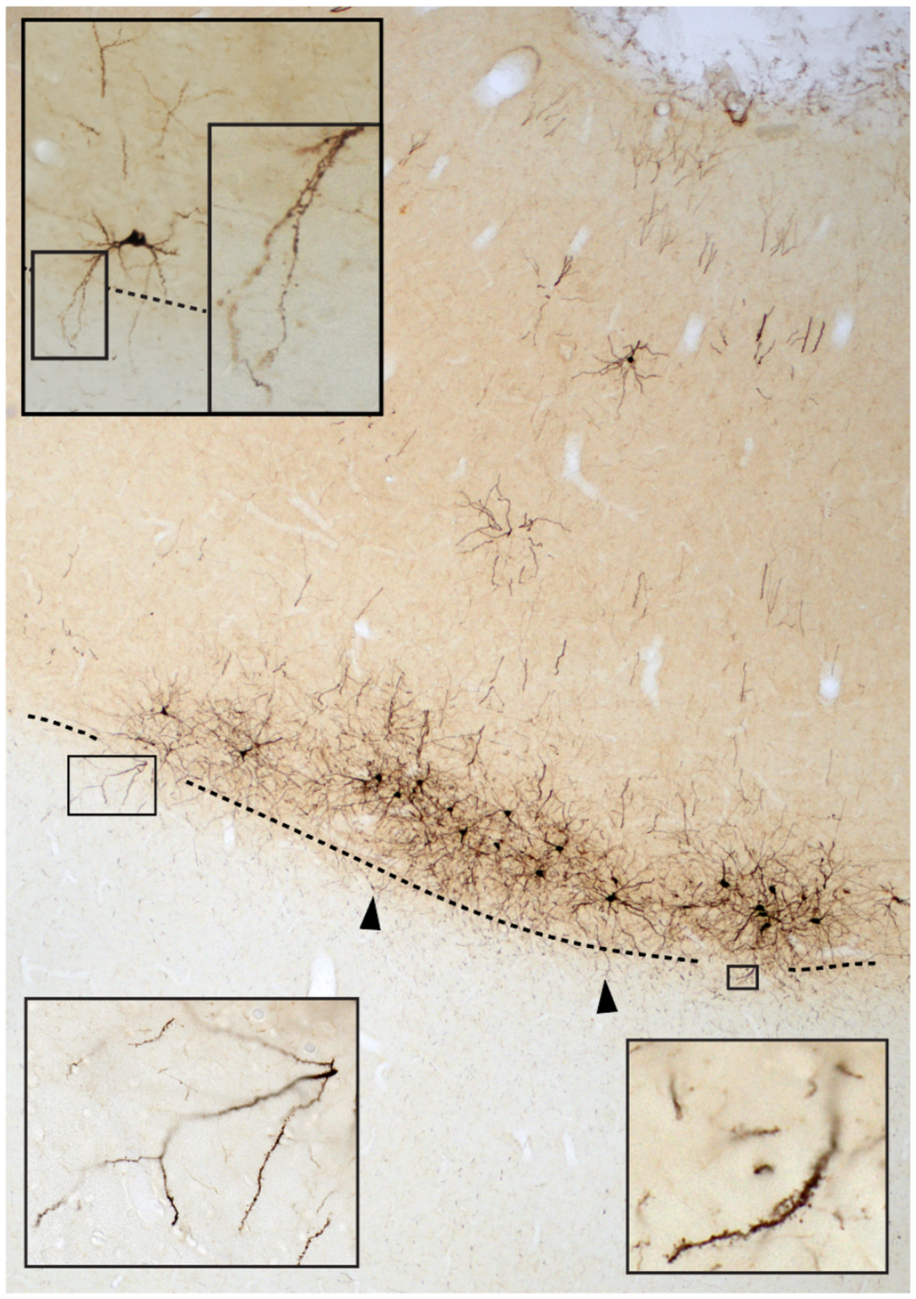
Photomicrograph of neurons retrogradely labeled by an injection of an adenovirus vector in area V2 of a macaque monkey. A dense field of neurons in layer 6 has dendritic incursion into subjacent white matter, indicated by the two arrowheads and two boxes (at low and higher magnification). Another example of an isolated layer 6 neuron from a separate field is shown at upper left, with the descending dendrite (boxed) at low and higher magnification. The dashed line corresponds to the WM-layer 6 border.

An important fact is that the WM, although cell-sparse, is not acellular and that there are abundant opportunities for synaptic and non-synaptic communication. First, there is a neurochemically and morphologically diverse population of phylogenetically conserved WM neurons. These neurons receive synapses (García-Marín et al., 2010), potentially engage in cross-talk with other WMNs and with axonal elements in the WM, and some participate in neurovascular regulation (reviews in Barbaresi et al., 2024; Fischer and Kukley, 2024). Second, there is extensive communication of myelinated axons, astrocytes and oligodendrocytes (e.g., Matute and Ransom, 2012, de Faria et al., 2021, Papanikolaou and Butt, 2017). Third (Figure 1), in the vicinity of cortical layer 6, there is extensive axonal and dendritic intermingling between GM and adjacent WM (Reveley et al., 2015). Further, the densely packed axonal environment may promote ephaptic coupling among axons (Debanne et al., 2011; Schmidt et al., 2021) and other modes of trans-axonal communication (e.g., Spead and Poulain, 2020). Taking these points into consideration, the common textbook version of separate compartments of cell-dense GM and axon-dense WM, increasingly seems unhelpful and inadequate.

This brief review of WM organization reexamines arguably outdated simplifications and assumptions (as stated in Bullock et al., 2022: "One challenge....is a reconsideration of what we actually know and what is instead a matter of convention"). We begin by briefly mentioning emerging views on the multiple roles of axon cytoskeleton and then revisit overlooked or debated points concerning WM organization from the neuroanatomical perspective, with direct attention to axon bundles or tracts. There is a large literature on WM and streamlines from a tractography point of view, with many excellent reviews addressing issues such as streamline density, orientation, and trajectory. For the sake of focus, we have mainly left this aside (but see reviews in Forkel et al., 2022; Jones et al., 2013; Mollink et al., 2017; Bullock et al., 2022; Yendiki et al., 2022; Zhang et al., 2022, and individual chapters in Dell'Acqua et al., 2025; Vavassori et al., 2025). Finally, we note that this review mainly focuses on the gyrencephalic primate brain. Comparisons between rodent brains and primate brains would need a separate review, but we note as one of the more dramatic differences that the WM in rodents is thin so that many of the extrinsic connections that in the primate use the white matter take an intra-cortical route in rodent layer 6 (Vandevelde et al., 1996; Yamashita et al., 2018).

## Intra-axon cytoskeleton

The axon is not a passive cable or wire (Debanne et al., 2011; Kirkcaldie and Collins, 2016; Alcami and El Hady, 2019). This fact is inescapably conveyed by even a casual inspection of the intricate nano-architecture with its complex functional interactions. The importance of the cytoskeleton is well known in the context of axonal transport (Grafstein and Forman, 1980), but as is increasingly apparent, it is also closely implicated in numerous metabolic and signaling processes. Many of these functions, particularly for what can be very long axons, are necessarily carried out to some extent autonomously of the cell body and/or in close cooperation with local glia ("an axon is therefore almost like a cell within a cell," Smith et al., 2023).

**Figure 2:**
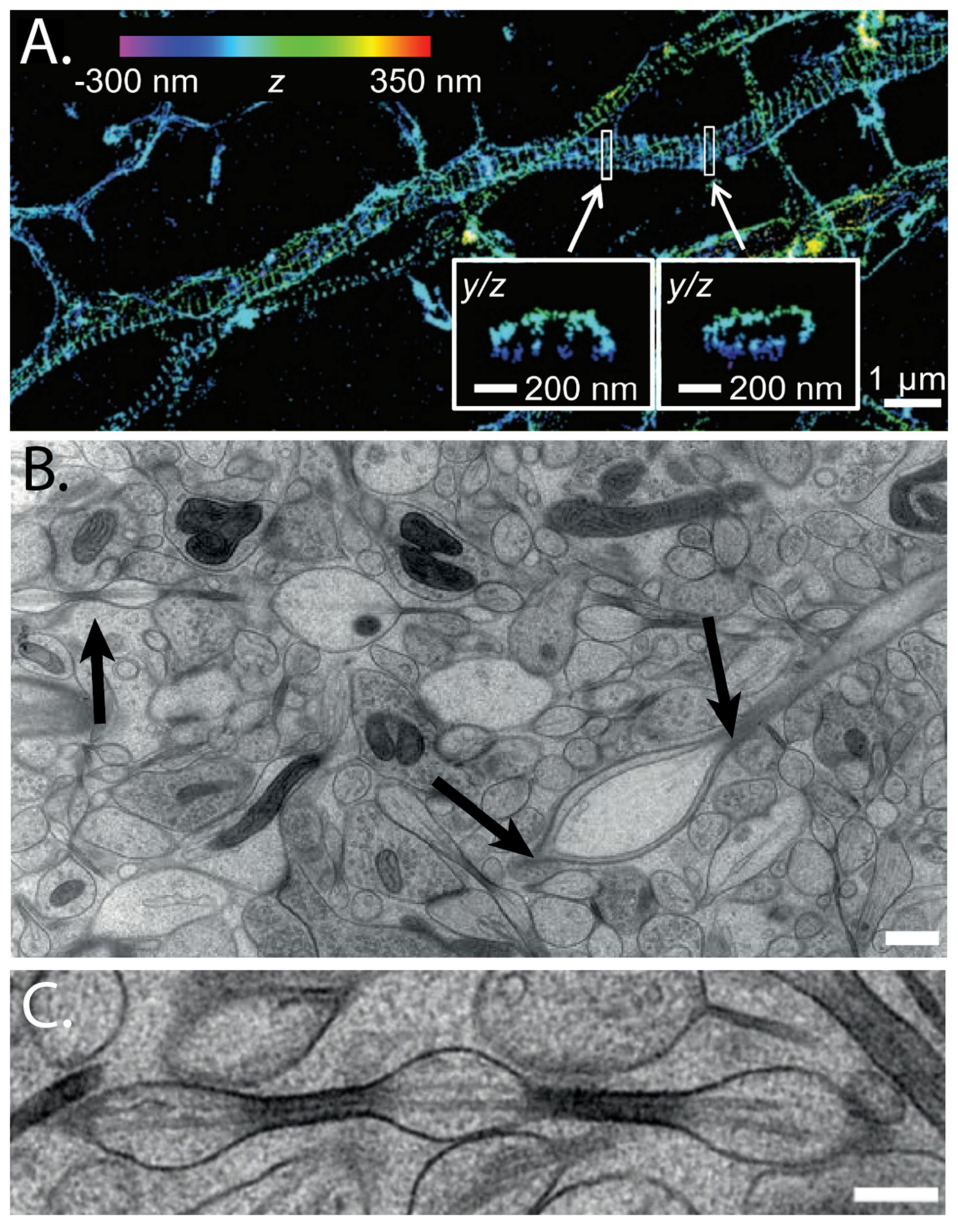
Axons have a complex cytoskeleton. **(A)** 3D STORM imaging reveals distinct organization of actin filaments, visualized as radial blue lines, by phallodion conjugated to Alexa Fluor 644. *YZ* cross-sections at the white rectangles are shown in the two higher magnification insets below. **(B)** Electron micrograph from acutely extracted mouse brain, with examples of pearled axon segments (arrows). Figure reproduced with permission from figure 1E in Xu et al., 2012. **(C)** Higher magnification image of axonal "pearls." Scale bars = 500 nm **(B)**, 200 nm **(C)** from figures 1a, b in Griswold et al., 2025.

Super-resolution microscopy and live imaging have brought to light previously unknown periodic structures such as actin rings and the alternating bands of spectrin (Xu et al., 2012; Leterrier et al., 2017), as well as deeper intra-axonal structures, such as dynamic actin assemblies comprised of focal "hotspots" at 3-4μm intervals and linear filamentous actin trails (Dubey et al., 2018). These structures are thought to function as flexible but supportive scaffolding elements, providing rigidity and stability to the elongated axon. The intricate and dynamic cytoskeleton may also be implicated in regulation of axon diameter and variability of diameter along an individual axon (Costa et al., 2018, 2020). More recently, repeating varicosities in unmyelinated axons that resemble "pearls on a string" (aka, " beading") have been discussed as a feature of axonal structure (Figure 2). Moreover, these structures are dynamically modulated by neuronal activity (Griswold et al., 2025), indicating that changes in activity have direct relations to axonal fine structure. Caliber variations and undulations, including "pearling," have discrete consequences for diffusion MRI signals (Andersson et al., 2020; Lee et al., 2024).

## Myelination

The myelin sheath is more than insulation (Fields, 2015). As is now well-established by electron microscopy, parameters such as myelin thickness vary substantially across related axons and even along an individual axon, sometimes with myelin-free gaps (Giacci et al., 2018; Lee et al., 2019).

Structural changes include modifications in internodes and internodal distribution, as visualized by long-term intravital myelin imaging and confocal microscopy (mouse somatosensory cortex: (Hill et al., 2018)), as well as variation in sheath number, length, and thickness (reviews in Hughes et al., 2018; Williamson and Lyons, 2018). Lifelong and activity-dependent myelin remodeling are associated with changes in functional plasticity (Fields, 2015; Xin and Chan, 2020; de Faria et al., 2021).

Another manifestation of myelin dynamics is the communication between an axon and its myelin-forming oligodendrocytes, what has been provisionally described as a putative axo-myelinic synapse Micu et al., (2018). That is, certain myelinated axons are proposed to secrete neurotransmitters in an activity-dependent manner, where corresponding receptors are activated on the inner myelin surface, thereby effecting an activity-dependent metabolic coupling between an axon and its myelin sheath.

## WM global organization

The global organization of cortical white matter can be discussed in several ways. The simplest is *descriptive, by spatial location* within the hemispheric volume and in relation to major ventricular and subcortical GM landmarks; for example, as descriptively named, the large fanned-out expanse of dorsal WM consists of the centrum semiovale (at levels dorsal to the lateral ventricle) and the immediately subjacent corona radiata Dejerine and Dejerine-Klumpke, (1895), (1901). These large WM territories contain axons exiting from and entering into the cortical GM but their descriptive designations do not typically address further subdivisions. The centrum ovale and corona radiata are specialized to large gyrencephalic brains. These and other of the many descriptive terms in the literature have been applied to smaller, lissencephalic brains, but then need some modifications. For example, portions of the "internal capsule" in the rodent brain pass through the striatum, whereas in the primate, the internal capsule passes between the caudate and putamen (e.g., Coizet et al., 2017).

A second approach highlights differences according the *length* of fiber trajectories. Early studies distinguished among short U-shaped intracortical association fibers (aka "*fibrae propria*" Meynert, 1885), intermediate and long association fibers, projection fibers and commissural fibers (see Schmahmann and Pandya, 2006). In current usage, "association" commonly refers to corticocortical axons and "projection" to axons directed to or coming from subcortical areas.

**Figure 3:**
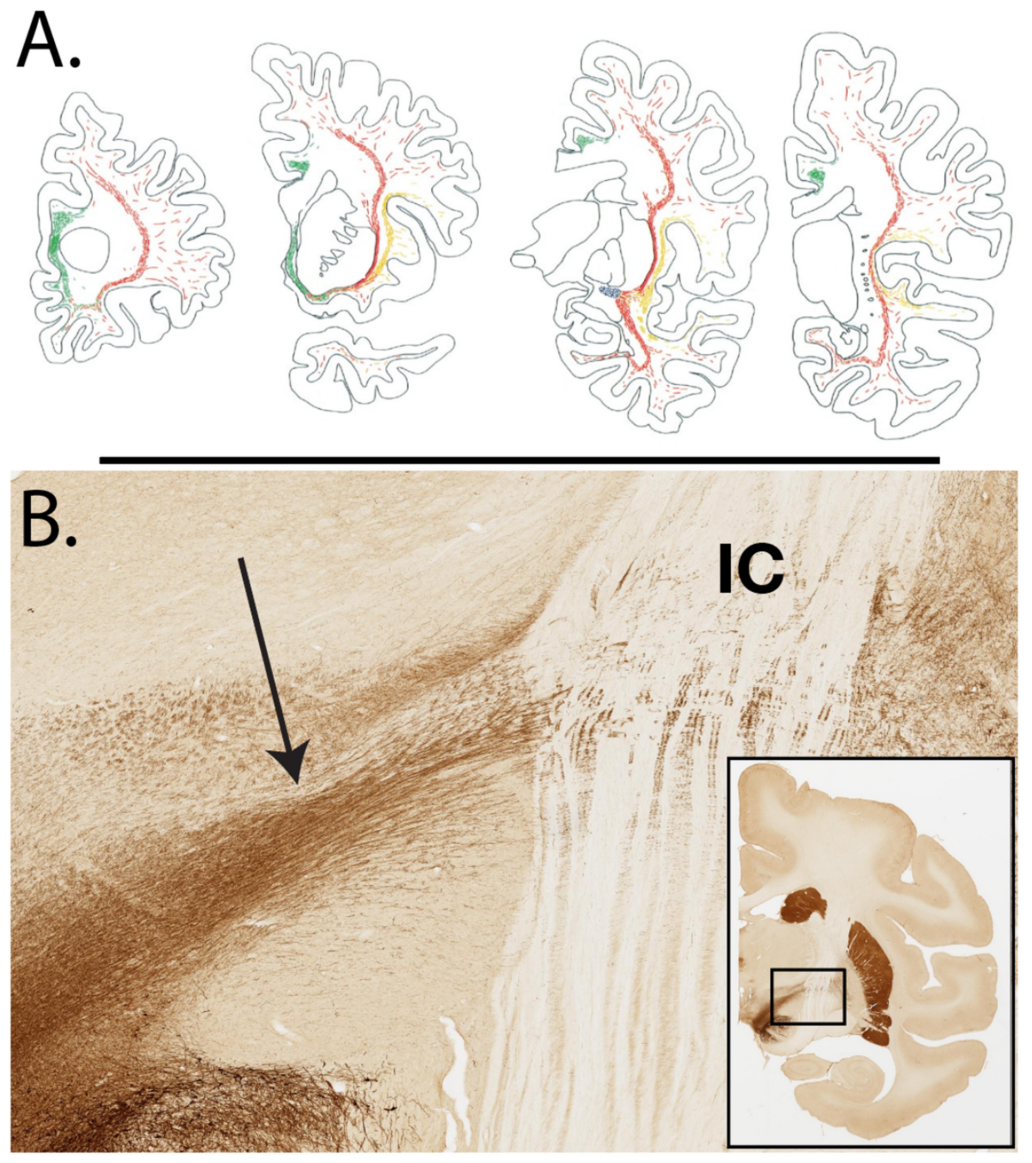
Some fiber bundles are neurochemically distinct. **(A)** AChE-rich (cholinergic) fiber bundles in the hemispheric WM of the human brain, in four coronal sections, anterior (at left) to posterior (at right). The medial cholinergic tract is depicted in green, and the two subdivisions of the lateral tract in red and orange. Reproduced with permission from Selden et al., 1998. **(B)** Tyrosine hydroxylase-rich fibers in the hemispheric WM of an 18.7-year-old rhesus macaque. Nigrostriatal fibers (arrow) are visualized, by immunocytochemistry and DAB, as they travel laterally toward and across the internal capsule (IC). The higher magnification view is from the boxed region in the low magnification coronal section inset at lower right. From Brain 124, section 33 in Macbrain Resource Center Collection 6 (https://macbraingallery.yale.edu/collection6/B124-TH/).

Another approach is by *neurochemical distinctions*. This has been extensively used in GM segmentation (e.g., Nimchinsky et al., 1996, 1997) but less so in WM. However, the major monoaminergic and cholinergic pathways are well-defined in primates (Figure 3; Selden et al., 1998). In primates (but not rodents), the thalamocortical projections are reliably visualized by parvalbumin or calbindin (figures 7D, E in Ding et al., 2016; Rockland, 2018). Neurotransmitter maps by acetylcholine, dopamine, noradrenaline, and serotonin immunocytochemistry or receptors are reported in the postmortem human brain (referred to as "neurochemical fingerprints," Zilles and Palomero-Gallagher, 2017; Hansen et al., 2022; Alves et al., 2025), and various rapid follow-ups specific to WM can be expected.

A *compartmental organization* of cortical and other GM zones has been well established both from neurochemical studies and connectivity studies in animal models; for example, cytochrome oxidase enzymatic compartments in primary and paraprimary visual areas in humans and nonhuman primates (NHP) (Livingstone and Hubel, 1984; Preuss et al., 1999; Sincich and Horton, 2005; Adams et al., 2007; Haenelt et al., 2023, among many others). Compartmental organization of WM has less of a history, but has been reported; for example, septa with alternating fiber orientations in the corpus callosum (≈200 μm wide, parvalbumin-defined in macaque, figure 6 in Rockland and Nayyar, 2012) and the "foliate" structure recently reported from MRI imaging of the human corpus callosum (Mollink et al., 2017; Wiggins et al., 2017; Lipp et al., 2024).

The most common approach to WM organization is according to discrete *axon bundles or tracts*. These tracts were first distinguished by gross anatomical observation and dissections (e.g., Dejerine and Dejerine-Klumpke, 1895, 1901; Ludwig and Klingler, 1956; Hau et al., 2017), often correlated with pathological alterations, and later corroborated by localized injections of tracer substances in animal models (e.g., Mufson and Pandya, 1984; Petrides and Pandya, 1984; Tusa and Ungerleider, 1985; Cavada and Goldman-Rakic, 1989; Ungerleider et al., 1989; Schmahmann and Pandya, 2006, among many others). Anatomically defined axon bundles are often further corroborated by en masse changes in fiber orientation, visualized by proxy Nissl stains or other markers (Axer et al., 2011a; Schurr and Mezer, 2021; Caspers et al., 2022); and see for example, the fronto-occipital fasciculus (figure 3-3 in Schmahmann and Pandya, 2006) and the optic radiations (Ludwig and Klingler, 1956; Rockland, 2018).

Although the overall global layout of white matter tracts is largely accepted (Bullock et al., 2022), important questions remain. How should a tract be conceptualized? Are tracts best defined anatomically in terms of origin and termination (“endpoints”) or as multi-component collections of fibers that partially share a common trajectory but vary in terms of their lengths, connections and network affiliation (discussed by Bajada et al., 2017, with reference to Dejerine; and van den Hoven et al., 2024)? How should we analyze the convergence of axons coming from separate tracts to the same target; for example, the convergence of commissural, thalamocortical, and corticocortical axons at a given target?

## Endpoints are not “points”

The tractwise approach (in anatomy or tractography) implicitly interprets “endpoints” as 2-way paired origins and terminations. This is a practical convention, and tracer studies in animals often adhere to this perspective, inasmuch as the experimental design usually involves injection *into* a defined origin (for anterograde tracers) or *into* a defined termination (for retrograde tracers) (reviewed in Yendiki et al., 2022, among others). Localized tracer injections in animal models allow mapping of tract subcomponents according to site of origin or termination, but interpretation requires multiple injections in the same animal or in different animals and is not routinely performed (but see: for the cingulum bundle, Heilbronner and Haber, 2014; for frontal cortex, Lehman et al., 2011; Morecraft et al., 2023).

A simple "pairwise" interpretation (area A projects to area B; one origin to one target), although a convenient convention, overlooks important complexities.

**Figure 4:**
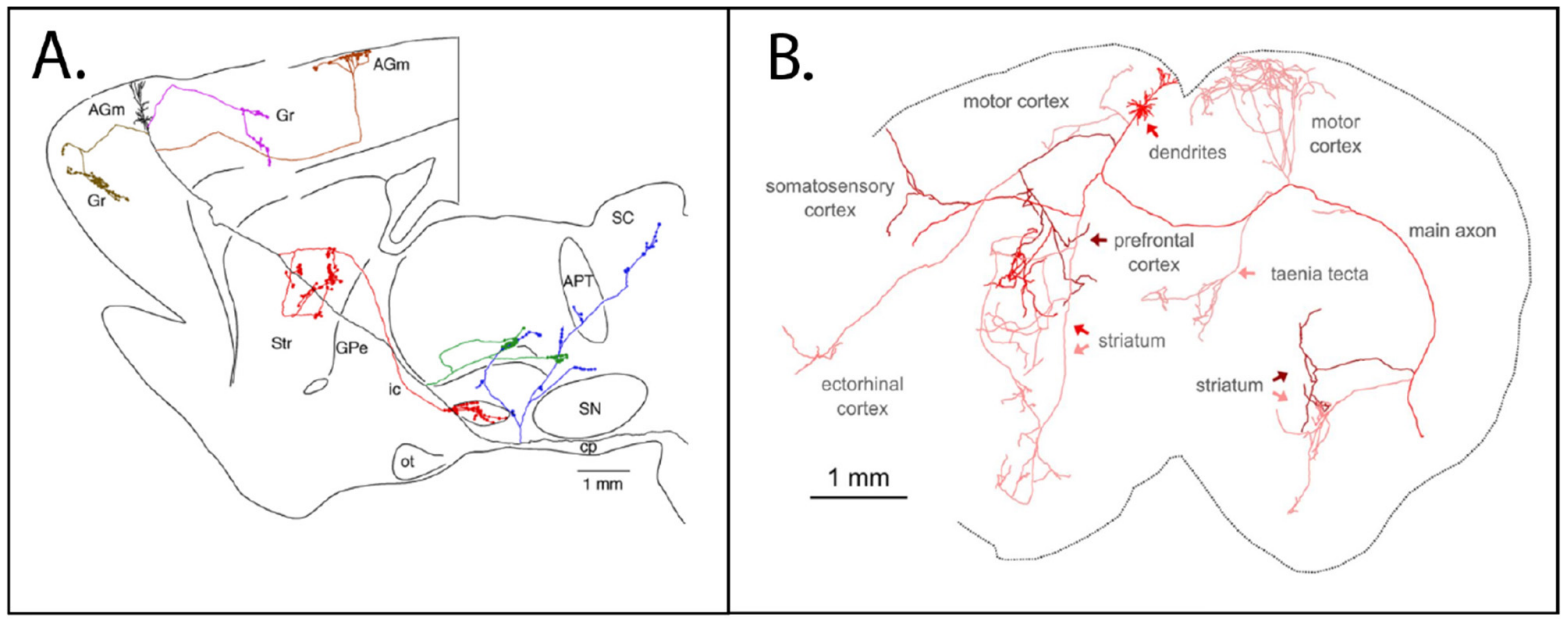
Cortical projection neurons have widespread axons with both intrinsic collateral branches and extrinsic collaterals diverging to multiple target structures. **(A)** Sagittal section schematic of a rat brain. A neuron with soma in the agranular motor cortex (AGm) has nearby intrinsic collaterals within AGm, in addition to a large number of collateral branches to extrinsic target structures. APT, anterior pretectal; GPe, globus pallidus, externa; Gr, granular; ic, internal capsule; IO, inferior olive; ot, optic tract; cp, cerebral peduncle; lfp, longitudinal fasciculus of the pons; py, medullary pyramid; SC, superior colliculus; SN, substantia nigra; Str, striatum. From figure 3A, Kita and Kita, 2012. **(B)** A neuron (arrow) in the mouse motor cortex has a widespread axon arborization to multiple cortical and striatal targets. Axonal segments are shaded to highlight arbors originating from common branch points. Whole axon reconstruction is overlaid onto a 2-D coronal histology section. From figure 6b, Economo et al., 2016.

**Figure 5:**
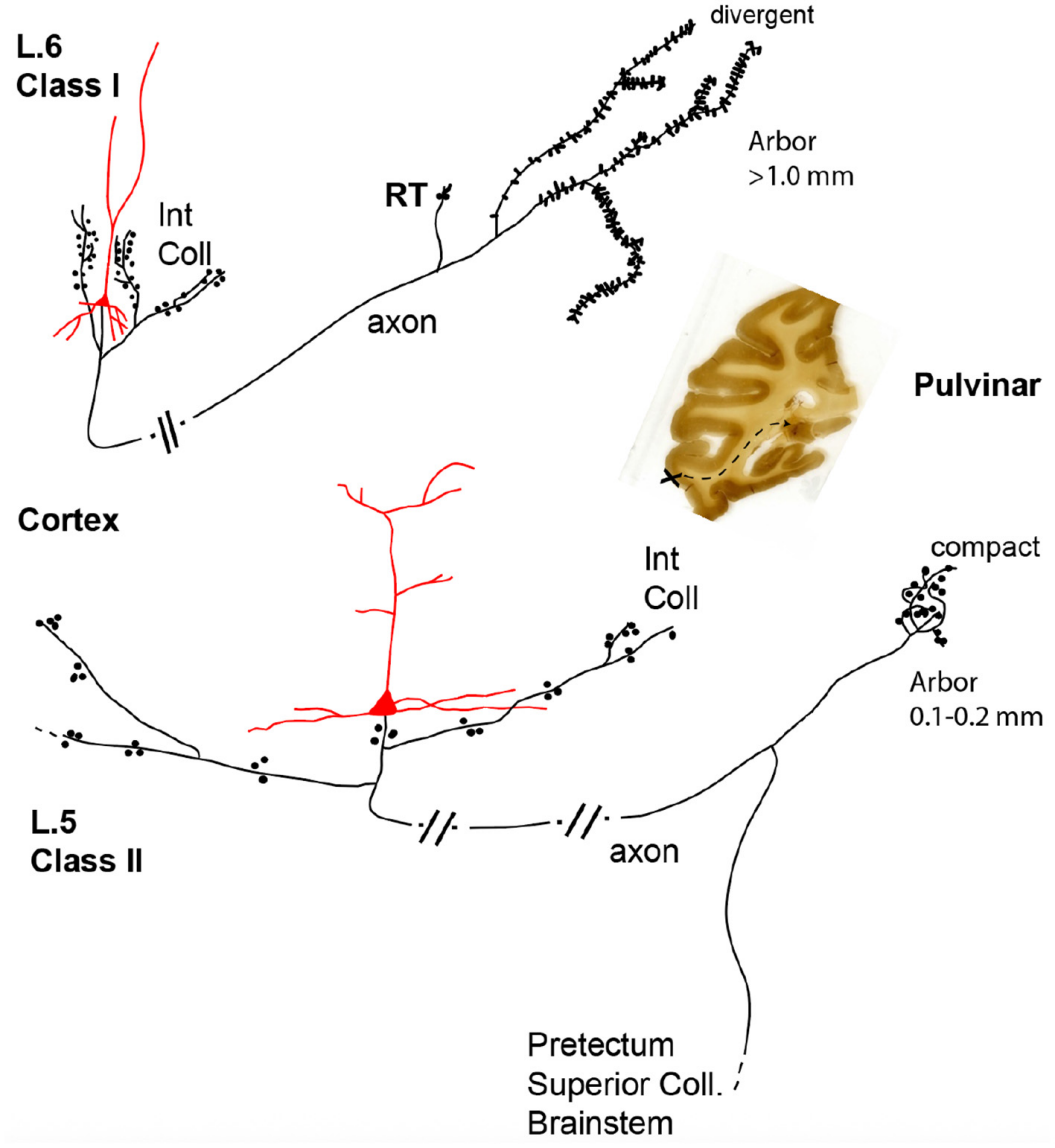
Projections from the same structure (here, a cortical visual area) *to* the same structure (here, thalamic pulvinar nucleus) can differ in multiple parameters. Cortical projections to the association thalamus (i.e., pulvinar; mediodorsal) differ in multiple respects. Those originating from layer 5 have extensive but bouton-sparse intrinsic collaterals (Int Coll), compact terminal arbors in the thalamus, and divergent extrinsic collaterals to additional subcortical targets. Those originating from layer 6 (in the same cortical area) have more local, bouton-dense intrinsic collaterals, divergent terminal fields in the thalamus, and further collaterals only to the reticular nucleus of the thalamus (RT). Originating corticothalamic neurons with typical dendrites are in red. Boutons are represented by dots (for the layer 5 neuron) or spinous protrusions (for the layer 6 neuron). As represented by parallel short lines along the axon, the full trajectory is only foreshortened and schematic. The coronal section at right shows the source (injected) area (at the X), and the corticopulvinar trajectory in schematic, by dashed lines. From figure 1 in Rockland, 2019.

1) Many neurons collateralize to multiple targets, as demonstrated in animal experiments by double injections of retrograde tracers (Fries et al., 1985; Perkel et al., 1986; Ugolini and Kuypers, 1986; Borra et al., 2010; Padberg et al., 2019, among many others), by anterograde axon visualization after intracellular fills or viral infection (Garraghty and Sur, 1990; Sato et al., 2000; Parent and Parent, 2006; Xu et al., 2021), and by the recently developed "barcode" technique (Kebschull et al., 2016; Han et al., 2018; Zeisler et al., 2024).

2) Most cortical neurons, in addition to one or more extrinsic projections, also have extended and extensive intrinsic collaterals (i.e., in the same local region as a designated parent soma). Consequently, a specific output target may receive *direct* input from a given neuron but additional convergent input from a wider set of neighboring neurons interconnected by intrinsic collaterals (Figure 4; Kita and Kita, 2012).

3) Distal terminations of an individual axon are not "pointwise" but rather diverge within a target area, often having 2-3 multiple arbors within a range of several millimeters (Rockland and Drash, 1996; Rockland and Knutson, 2000). Moreover, the same volume of cortex can spatially map in different ways onto the same target; for example, cortical projections to association thalamus. That is, layer 5 neurons use focal thalamic terminations, while layer 6 neurons in the same tissue volume have terminations over much wider extents in the same thalamic field (Figure 5; Rockland, 2019).

The “endpoints” terminology tends to obscure the fact of distal divergence within a GM target, as well as the collateralization of one neuron to multiple targets (point 1, above).

## Topography: a convenient term but complicated reality

Orderly topographic organization has long been a hallmark in GM investigations, which commonly report brain regions as having a convincing representation of external sensory or motor coordinate systems (e.g., Petersen et al., 2024). Earlier results from pathological lesions in human patients supported the idea that orderly GM topography continues through the trajectory of fiber tracts to the terminal target structure (Bumke and Foerster, 1936; Hardy et al., 1979; Tredici et al., 1982); and localized tracer injections in animal models appeared to corroborate some degree of spatial segregation in certain white matter regions (see Schmahmann and Pandya, 2006, among many other investigators). For example, prefrontal cortical (PFC) tracer injections in macaque monkeys (Lehman et al., 2011) reveal spatial differences in axons exiting from different ventral PFC areas, where axons from medial regions travel ventral to those from more lateral areas. Corticothalamic fibers are situated dorsal to those going to the brainstem. Comparably detailed data are sparse however, even for the early sensory pathways and cortices.

**Figure 6:**
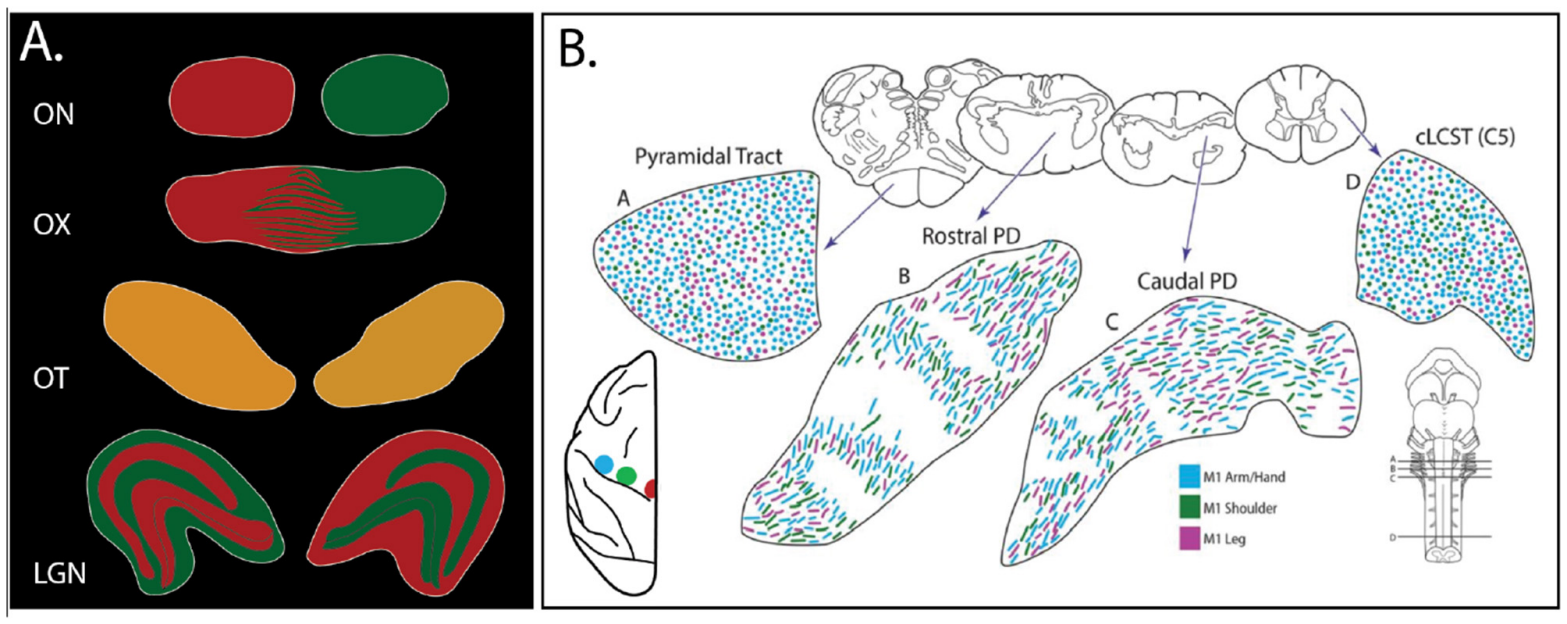
"Togography" is complicated in reality. **(A)** Cartoon schematic to illustrate the changing retinal fiber arrangement from segregation, proximally in the optic nerve (ON), to intermingling, more distally in the trajectory through the optic chiasm (OX) and optic tract (OT), to the termination (again segregated) in the lateral geniculate nucleus (LGN). Green represents injection of Alexa fluor 488 (in the right eye) and red represents injection of Alexa fluor 594 in the left eye (of macaque monkey). The fluorescent signal appears yellow when fibers from the two eyes are finely interspersed, as happens posterior to the optic chiasm (see figure 1 in Horton et al., 2023). **(B)** In the distal portion of the corticospinal tract (in the pons and spinal cord), the topographic organization according to body part is scrambled. A–D: four selected anterior-to-posterior coronal sections from a macaque monkey (corresponding low magnification insets above, for the higher magnification plots below) of axons differentially labeled from the injection sites shown in the schematic cerebral hemisphere at lower left (color matched for injection sites and resultant labeled terminations). Coronal section levels are shown on the gross brainstem at lower right and modified with permission from figure 7 in Lemon and Morecraft (2023).

A more complicated interpretation of topographic organization is suggested by recent studies clearly showing that topography may be significantly altered along the trajectory of a given tract (Saunders and Aggleton, 2007; Horton et al., 1979; Lemon and Morecraft, 2023). Traditional views of the well-studied optic tract are significantly questioned by the results of separate tracer injections in the left and right eyes of macaque monkeys (Figure 6). At the optic chiasm proper, colorimetrically distinct tracers injected in each eye resulted, as expected, in segregated sheets of green- or red color-distinguished axons from the two eyes, but posterior to the chiasm, crossed fibers ‘completely intermingled' with the uncrossed fibers (Naito, 1994; Horton et al., 2023; Pawar et al., 2024).

The corticospinal tract is another example of an apparently scrambled topography (Figure 6). In classic depictions, axons in the descending pathway are shown as organized in a body-recognizable homunculus. From close investigation with distinguishable tracer injections in macaque motor cortex, however, labeled axons from spatially separate sites are seen as intermingled, not segregated according to body map, especially toward their distal terminations in the brainstem and spinal cord (Lemon and Morecraft, 2023; Morecraft et al., 2023). Moreover, along a defined trajectory, there can be multiple steps of fasciculation, defasciculation, and refasciculation; for example, dopaminergic and serotonergic fibers en route to the basal ganglia (Wallman et al., 2011).

The "scrambling" of axon position in fiber tracts can seem puzzling and raises the question of how axons lose and then apparently re-establish order. Is axon position less tightly tethered to the parent neuron than has usually been understood ("axon autonomous," Smith et al., 2023)? A useful insight comes from developmental studies, which have distinguished populations of "pioneer" and "follower" axons. Pioneer axons follow stricter routes, while follower axons have looser trajectories (e.g., O'Leary and Koester, 1993; Franze, 2020; Dumoulin and Stoeckli, 2023). It is also worth noting that pathway development is shaped by gradients of site-specific diffusible chemoattractants and chemorepellants, which can stabilize some axons while pruning others (Stoeckli, 2018; Spead and Poulain, 2020; Breau and Trembleau, 2023). These processes may influence defasiculation, allowing initially bundled axons to change position in response to local cues during development (Sitko and Mason, 2016; Weaver and Poulain, 2021).

Several additional factors might account for the more nuanced results on topographic organization. One is that the classical pathway origin itself has a more complex spatial organization that may be better described as multiple functionally distinct regions rather than as a single topographic map (Geyer et al., 1996; Binkofski et al., 2002; Rathelot and Strick, 2009; Deo et al., 2024). Thus, in the case of motor cortex, high resolution fMRI methods report a spatial interdigitation of action control-linked and motor effector regions (Gordon et al., 2023; and see "spotlight," Graziano, 2023: "the motor homunculus is fundamentally wrong," a conclusion based in part on data using the extended stimulation method, with a timescale similar to that of meaningful behavior). Cortico-motoneuronal cells labeled from retrograde transneuronal transport of rabies virus from injections in single muscles have been found in overlapping and intermingled patterns, more consistent with a wide variety of muscle synergies than with any focal body part representation (Rathelot and Strick, 2006). In the visual system, a supra-areal organization based on visual eccentricity is considered a possible extension to the discrete mappings of individual cortical areas (Arcaro and Kastner, 2015).

A second factor, relevant to "scrambling" along a fiber trajectory, is that projections frequently originate from a distributed set of GM areas. Retrograde tracer injections in the macaque spinal cord demonstrate a distributed pattern of corticospinal source neurons, from multiple areas besides motor cortex (Dum and Strick, 1991; Galea and Darian-Smith, 1994; Rozzi et al., 2006). How these co-terminate and recombine in a single target is unclear.

## Technical progress

The recent surge in technological developments has direct applicability to WM investigations. Improvements in label-free polarized light imaging (Axer et al., 2011a,b) are better addressing the joint needs of high resolution and large field visualization that have been so challenging in human brains. The current iteration, ComSLI ("computational scattered light imaging") uses a rotating LED light source and high-resolution camera to visualize multiple crossing fiber orientations per image pixel (Heiden et al., 2024; Georgiadis et al., 2025). Large spatial scale, high resolution brain mapping has also been achieved by multiple studies using different protocols for tissue optical clearing and 3-D fluorescence microscopy (e.g., Sorelli et al., 2025; review in Ueda et al., 2020). Expansion-assisted selective plane illumination microscopy (ExA-SPIM) aims to bridge the micro- and macro-scales of brain connectivity by tracing individual axons in densely packed white matter, scalable over long distances ("axonal connectomics," Glaser et al., 2023; Takasaki et al., 2025). For further discussion and review of other approaches, see the reference list in the representative publications listed above.

Single axon analysis, with the increasing availability of reconstruction algorithms (Sundaresan et al., 2025), is becoming more routine with much larger sample sizes, ranging from dozens to hundreds or even thousands of examples. Tracer injection-based results largely confirm older results on the heterogeneity of target divergence (e.g., Coudé et al., 2018, Table 1; and see above), but provide further detail regarding trajectories and characterization of the parent neuron. There is already a large database of high resolution reconstructions in mice (Winnubst et al., 2019; Liu et al., 2025), and multiple reports have now been published in NHP (Xu et al., 2021; Gou et al., 2025). Comparable high resolution brainwide data from human brains will be more challenging.

## Conclusion

**Figure 7:**
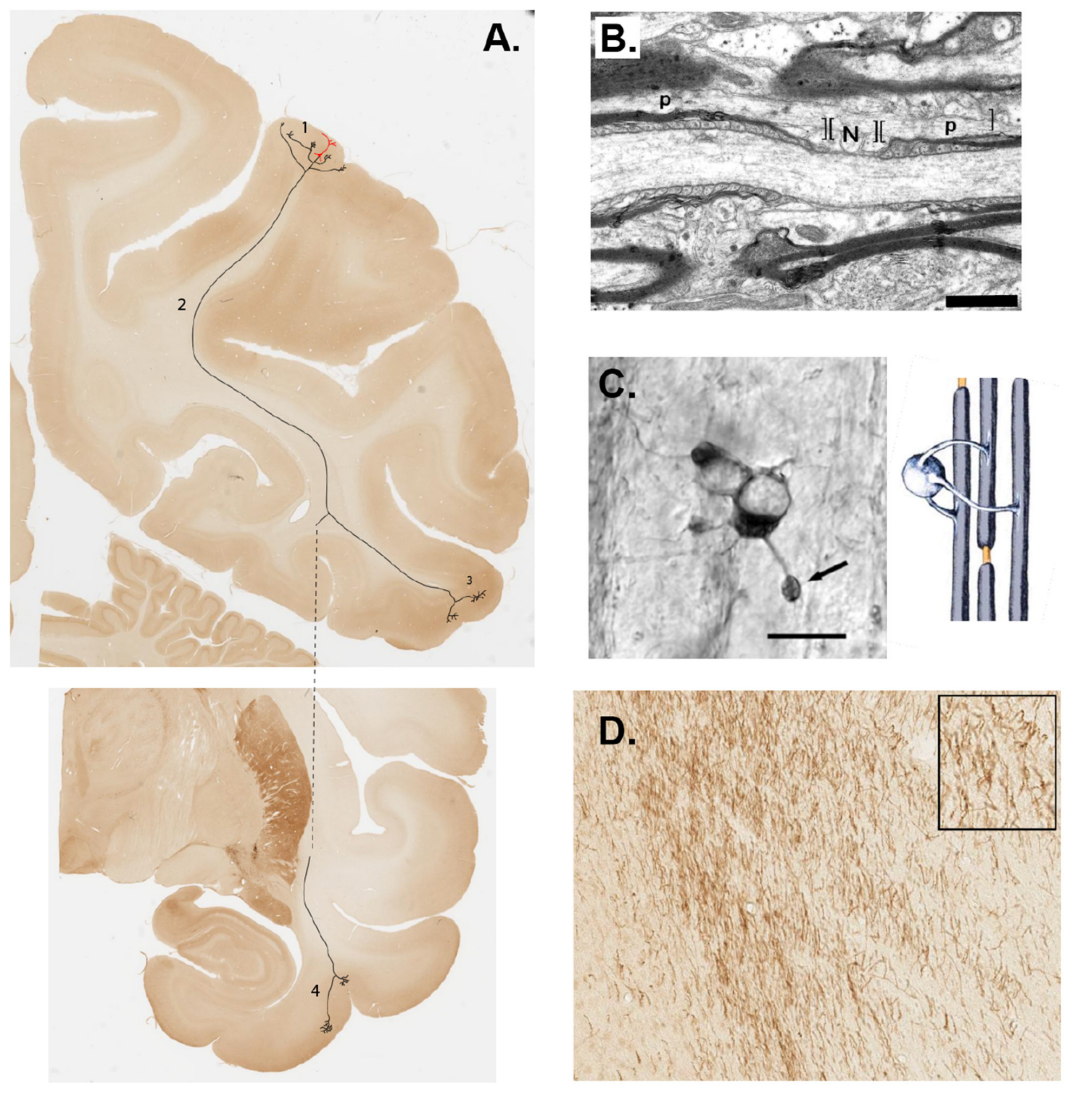
Brain communication proceeds simultaneously over several spatial levels. **(A)** A cortical projection neuron (soma and dendrites in red) is superimposed on a coronal section (macaque monkey) to represent 1) local collaterals, 2) axon trajectory (dashed line to depict anterior traverse in the z-dimension), and 3) extrinsic cortical terminations (target 1). Coronal section (cropped image) at the bottom represents 4) a second, anteriorly distant terminal field (target 2). **(B)** Longitudinal section of a myelinated axon in the primary visual cortex of a rhesus monkey. N = node of Ranvier, as bracketed by paranodes (p). Scale bar = 1.0 μm. **(C)** Light microscopic image of an oligodendrocyte with multiple myelinating processes in association with different axons (Scale bar = 10 μm). At right of **(C)** diagrammatic representation of an oligodendrocyte and its myelinated group of axons. **(B, C)** are reproduced from Peters (2009), figures 1, 4, and 5. **(D)** Cholinergic axons labeled by ChAT antibody, in the cingulum bundle, as these approach their target in the cingulate cortex. The higher magnification inset shows individual axons. From section 18, Brain 89, 6.2-month-old macaque monkey (https://macbraingallery.yale.edu/collection6/#B89).

In this selective review, we set out to emphasize several points where there has been notable progress in views of WM organization at different scales (i.e., axon nano-architecture to bundles), as well as several points that have prompted revision of older views (e.g., topography). In many respects, the study of WM has lagged behind investigation of the better analyzed and mapped GM. It seems likely that this situation will change. The advantages of a more holistic approach that encompasses features of source neurons, axon trajectories and branching, and multiple postsynaptic targets are obvious and compelling (Figure 7). Brainwide spatial localization is needed for better characterization of the coordinated functioning and distributed activity changes across the WM, and of the communication modes utilized by WM in conjunction with GM regions (for GM see, for example, Mohar et al., 2025). In the immediate future, we can expect new results and new assays with relevance to a broad range of questions, such as variability (lifespan, individual, and species-specific), plasticity effects, and white matter remodeling under different conditions, including therapeutic interventions (e.g., Fujimoto et al., 2024). These advances will bring WM investigation more in line with the multi-scale approaches, from subcellular to supracellular and networks, which have been successfully adopted in GM investigations. A more realistic view will see WM not as secondary to and remote from GM, but anatomically and functionally integrated with GM, recognized as an obligatory partner.
